# Curcumin: A review of anti-cancer properties and therapeutic activity in head and neck squamous cell carcinoma

**DOI:** 10.1186/1476-4598-10-12

**Published:** 2011-02-07

**Authors:** Reason Wilken, Mysore S Veena, Marilene B Wang, Eri S Srivatsan

**Affiliations:** 1 Department of Surgery, VA Greater Los Angeles Healthcare System, West Los Angeles, CA, USA; 2 Division of Head and Neck Surgery, David Geffen School of Medicine at University of California Los Angeles, Los Angeles, CA, USA; 3 Department of Surgery, David Geffen School of Medicine at UCLA, Los Angeles, CA, USA

## Abstract

Curcumin (diferuloylmethane) is a polyphenol derived from the *Curcuma longa* plant, commonly known as turmeric. Curcumin has been used extensively in Ayurvedic medicine for centuries, as it is nontoxic and has a variety of therapeutic properties including anti-oxidant, analgesic, anti-inflammatory and antiseptic activity. More recently curcumin has been found to possess anti-cancer activities via its effect on a variety of biological pathways involved in mutagenesis, oncogene expression, cell cycle regulation, apoptosis, tumorigenesis and metastasis. Curcumin has shown anti-proliferative effect in multiple cancers, and is an inhibitor of the transcription factor NF-κB and downstream gene products (including c-myc, Bcl-2, COX-2, NOS, Cyclin D1, TNF-α, interleukins and MMP-9). In addition, curcumin affects a variety of growth factor receptors and cell adhesion molecules involved in tumor growth, angiogenesis and metastasis. Head and neck squamous cell carcinoma (HNSCC) is the sixth most common cancer worldwide and treatment protocols include disfiguring surgery, platinum-based chemotherapy and radiation, all of which may result in tremendous patient morbidity. As a result, there is significant interest in developing adjuvant chemotherapies to augment currently available treatment protocols, which may allow decreased side effects and toxicity without compromising therapeutic efficacy. Curcumin is one such potential candidate, and this review presents an overview of the current in vitro and in vivo data supporting its therapeutic activity in head and neck cancer as well as some of the challenges concerning its development as an adjuvant chemotherapeutic agent.

## Introduction

Head and neck squamous cell carcinoma (HNSCC) is the sixth most common form of cancer worldwide and represents approximately 5% of all cancers diagnosed annually in the United States [1,2]. Every year more than 30,000 cases of oral and pharyngeal cancer are diagnosed, and over 8,000 individuals die of the disease. If the definition of HNSCC is expanded to include laryngeal cancers, the number of annually diagnosed cases grows to over 42,000 individuals and results in over 12,000 deaths per year in the United States [3].

The development of head and neck malignancies is strongly associated with certain risk factors such as tobacco use, alcohol consumption and environmental exposures (such as EBV infection in the case of nasopharyngeal carcinoma). Additionally, human papilloma virus (HPV) infection is emerging as a causative agent for HNSCC and may account for the increased incidence of this malignancy in patients who do not have histories of alcohol or tobacco usage [4]. By virtue of their inconspicuous location, many cases of HNSCC are not discovered until the cancer is at a later stage, not uncommonly until after spread to lymph nodes in the neck. A growing tumor in the head and neck region may not cause any discernable symptoms and a large portion of earlier-stage oral tumors are discovered incidentally, often by dental providers.

Standard treatment regimens for head and neck cancer depend on the stage of the disease. Early stage (stage I and II) tumors are treated primarily with surgery or radiotherapy, with both modalities resulting in similar local control and survival rates. Radiation may also be used postoperatively when surgical margins are close or positive, or if perineural or lymphovascular invasion by tumor is found. More advanced (stage III and IV) cancers often require multi-modality therapy with surgery, radiation and chemotherapy which can result in very high morbidity [5]. Concurrent chemoradiation (CRT) for locoregionally advanced HNSCC is the preferred treatment in cases of unresectable disease or instances when surgical resection would result in an unacceptable functional loss for the patient. Recent years have seen a trend towards organ-sparing therapies of chemotherapy and radiation for resectable cancers of the oropharynx, hypopharynx and larynx [6].

Platinum-based agents form the backbone of the standard chemotherapeutic regimens for head and neck cancer. Cisplatin (cis-diamminedichloroplatinum) is a widely used drug in the class of platinum-based chemotherapies (which also includes carboplatin & oxaliplatin). The platinum compounds work by the formation of DNA crosslinks within cells, leading to apoptosis and cellular senescence. The efficacy of cisplatin in HNSCC is greatly increased when combined with other chemotherapeutic agents, such as taxanes (paclitaxel and docetaxel) and 5-fluorouracil (5-FU) (Table 1) [7-10].

**Table 1:** Current chemotherapeutic models in head and neck cancer

Agent	Mechanism	Mode of Usage in HNSCC	Significant Adverse Effects	Reference
Platinum agents (Cisplatin/Carboplatin)	Formation of DNA adducts, induction of apoptosis and senescence	Cisplatin/Carboplatin single-agent Cisplatin/Carboplatin in combination with 5-Flurouracil	Nephrotoxicity (acute renal failure, chronic renal insufficiency) Ototoxicity (high-frequency hearing loss) Neurotoxicity (peripheral neuropathy) Hematologic (myelosupression) Gastrointestinal (nausea, vomiting) Electrolyte disturbances (hypomagnesiumia/hypokalemia/ hypocalcemia)	[8-12]
5-Flurouracil	Anti-metabolite (pyrimidine analog, inhibits thymidylate synthase)	5-Flurouracil in combination with Cisplatin/Carboplatin, and with or without Paclitaxel	Cardiac toxicity (angina, myocardial ischemia), Gastrointestinal (nausea, omiting, ulcers), Hematologic (myelosupression), Thrombophlebitis, Dermatologic (rash)	[8-10]
Taxanes (Paclitaxel/Docetaxel)	Anti-microtubular agent (inhibitor of mitosis)	Paclitaxel in combination with Carboplatin/Cisplatin, and with or without 5-Fluoruracil	Cardiovascular (hypotension, EKG changes) Gastrointestinal (mucositis, nausea, vomiting) Hematologic (neutropenia, leukopenia, thrombocytopenia), Neuromuscular (peripheral neuropathy, myalgias), Hepatic (elevated liver enzymes)	[9,10]
Cetuximab	Anti-EGFR monoclonal antibody	Cetuximab plus radiation therapy Single-agent cetuximab in platinum refractory HNSCC Phase I/II trial of cetuximab plus 5-FU and platinum agents (on going)	Infusion reaction Dermatologic (acneform rash, pruritis) Gastrointestinal (abdominal pain, constipation, diarrhea, nausea, vomiting), Respiratory (dyspnea, cough), Neuromuscular weakness	[14-17,202]

The potential adverse effects from the treatment for HNSCC are numerous. Radical surgery can result in disfigurement & functional impairment, but even the organ-preserving treatment methods of radiation and chemotherapy may result in a host of negative side effects, some permanent [11]. Common side effects of radiotherapy include mucositis, oral candidiasis, loss of taste and xerostomia, which may be permanent due to the damaging effect of radiation on the salivary glands [11]. Osteoradionecrosis of bones within the radiation field (most commonly the mandible) may occur as a result of damage to the bone vasculature and osteocytes and is one of the most serious complications of radiotherapy [12]. The toxic effects of platinum-based chemotherapy are dose dependent and include renal, otologic, and bone marrow suppressive sequelae (Table 1) [7-10].

Despite continuing research and advances in treatment, the clinical outcomes and overall survival rates for HNSCC have not improved significantly over the last several decades, with the overall 5 year survival rate as low as 50% [1,2,13]. As a result, there has been continuing investigation into potential alternative and less toxic therapies for head and neck cancer, with the aim of achieving a more favorable clinical outcome while reducing treatment morbidity. The class of molecularly targeted therapies against the epidermal growth factor receptor (EGFR) is one such example, as EGFR is overexpressed in a number of head and neck cancers. Cetuximab is an anti-EGFR monoclonal antibody that was approved by the Food and Drug Administration in 2004 for the treatment of advanced colon cancer. In 2006, Cetuximab was approved for use in head and neck squamous cell carcinoma---both in combination with radiotherapy for advanced HNSCC as well as single-agent therapy for platinum-refractory head and neck cancer (Table 1). Several studies of cetuximab as an adjuvant agent with radiotherapy have demonstrated improved locoregional control and statistically significant increases in both progression-free and overall survival [14,15]. The addition of cetuximab to standard platinum-based chemotherapy in platinum-resistant recurrent or metastatic head and neck cancer has also been studied and demonstrated increased treatment efficacy and improved overall survival without a significant increase in toxicity [16,17].

Curcumin, an Indian spice with antioxidant, anti-inflammatory and anti-cancer properties, has shown promise both as a potential chemopreventive agent as well as a novel adjuvant treatment for head and neck malignancies. This review will discuss the biological properties of curcumin, with emphasis on the molecular pathways modulated by the spice that may prove useful in the treatment of head and neck squamous cell carcinoma.

### Curcumin

**Figure 1:**
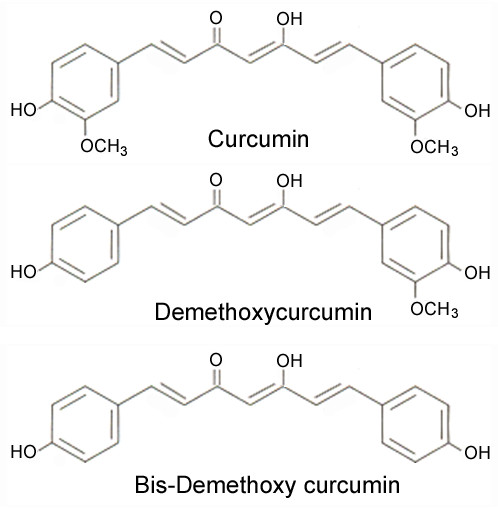
**Structure of the curcuminoids curcumin, demethoxycurcumin and bisdemethoxycurcumin**.

Curcumin (diferuloylmethane) is the chief component of the spice turmeric and is derived from the rhizome of the East Indian plant *Curcuma longa*. *Curcuma longa *is a member of the Zingiberacae (ginger) family of botanicals and is a perennial plant that is native to Southeast Asia [18]. Turmeric contains a class of compounds known as the curcuminoids, comprised of curcumin, demethoxycurcumin and bisdemethoxycurcumin (Figure 1) [19]. Curcumin is the principal curcuminoid and comprises approximately 2-5% of turmeric; it is responsible for the yellow color of the spice as well as the majority of turmeric's therapeutic effects [18]. Aside from being employed as a flavoring and coloring agent in food, turmeric has also been widely used in Ayurvedic medicine for its anti-oxidant, antiseptic, analgesic, antimalarial and anti-inflammatory properties [20]. Curcumin has been consumed as a dietary supplement for centuries and is considered pharmacologically safe [21].

### Antioxidant Activity

Curcumin is a lipophilic polyphenol and thus is insoluble in water, but is readily soluble in organic solvents such as dimethylsulfoxide, acetone and ethanol [20,22]. The antioxidant activity of the curcuminoids comes by virtue of their chemical structure. The curcuminoids consist of two methoxylated phenols connected by two α, B unsaturated carbonyl groups that exist in a stable enol form [23]. Curcumin has been shown to inhibit lipid peroxidation using linoleate, a polyunsaturated fatty acid that is able to be oxidized and form a fatty acid radical. It has been demonstrated that curcumin acts as a chain-breaking antioxidant at the 3' position, resulting in an intramolecular Diels-Alder reaction and neutralization of the lipid radicals [24]. In addition to inhibiting lipid peroxidation, curcumin demonstrates free radical-scavenging activity. It has been shown to scavenge various reactive oxygen species produced by macrophages (including superoxide anions, hydrogen peroxide and nitrite radicals) both in vitro as well as in vivo using rat peritoneal macrophages as a model [25,26]. Inducible nitric oxide synthase (iNOS) is an enzyme found in macrophages that generates large amounts of NO to provide the 'oxidative burst' necessary for defense against pathogens. iNOS is induced in response to an oxidative environment, and the NO generated can react with superoxide radicals to form peroxynitrite, which is highly toxic to cells. It has been shown that curcumin downregulates the iNOS activity in macrophages, thus reducing the amount of reactive oxygen species (ROS) generated in response to oxidative stress [27,28]. Additional studies in microglial cells (brain macrophage analogs) demonstrated reduced NO generation and protection of neural cells from oxidative stress following curcumin treatment, thus the spice and may be useful in reducing the neuroinflammation associated with degenerative conditions such as Alzheimer's disease [29-31].

### Anti-inflammatory Activity: Effect on NF-κB Pathway

Curcumin has been shown to suppress the activation of NF-κB, an inducible transcription factor that regulates the expression of a host of genes involved in inflammation, cellular proliferation and cell survival [32-57]. Genes regulated by NF-κB include cyclooxygenase-2 (COX-2), IκBα, TNF-α, cyclin D1, ICAM-1, c-myc, Bcl-2, MMP-9, inducible nitric oxide synthase (iNOS), and interleukins including IL-6 and IL-8 [19,35,36,58,59]. NF-κB expression is involved in cellular responses to stressful stimuli such as cytokines, UV irradiation, free radicals, hypoxia (including cigarette smoke) and infectious antigens [60-64]. Activation of NF-κB is increased in many cancers, and is associated with various steps in the development of malignancy such as expression of anti-apoptotic genes, angiogenesis, tumor promotion and metastasis [65]. Studies from our laboratory as well as others have demonstrated constitutive expression of NF-κB in head and neck squamous cell carcinoma [45,48,53,66,67].

NF-κB is a heterodimeric protein composed of five subunits: RelA (p65), RelB, c-Rel, NF-κB1 (p50 and p105) and NF-κB2 (p52) [68]. The complex is retained in the cytoplasm in an inactive form by IκB (inhibitor of NF-κB), which is composed of α and β subunits. Upon receipt of the appropriate chemical signals that initiate NF-κB activation, several steps are required to free NF-κB from this inhibitory binding. IκB must be phosphoylated at it's α subunit by IκK (inhibitor kappa B kinase) which results in ubiquitination and degredation of the phosphorylated IκBα and the release of NF-κB from its stationary location in the cytoplasm. The unbound NF-κB is then available for transport into the nucleus where it may bind to DNA and activate transcription.

Curcumin's inhibitory effect on the NF-κB pathway is central to providing the compound with its anti-inflammatory properties. Curcumin blocks the IκK-mediated phosphorylation and degredation of IκBα, thus NF-κB remains bound to IκBα in the cytoplasm and is not able to enter the nucleus to activate transcription [34,35,53]. Studies involving suppression of NF-κB activity have demonstrated a subsequent down-regulation of COX-2 and iNOS and decreased production of inflammatory markers [33,36,41]. Consistent with its effect on NF-κB, curcumin has been shown to inhibit the production of inflammatory cytokines by activated monocytes and macrophages, including COX-2, liopxygenase (LOX), iNOS, monocyte chemotactic protein-1 (MCP-1), monocyte inflammatory protein-1 (MIP-1α) and interleukins including IL-1, -2, -6, -8, and -12 [69,70].

In addition to inhibiting NF-κB activation, curcumin also has suppressive effects on other pathways involved in inflammation. The arachadonic acid pathway for eicosanoid biosynthesis is an important participant in the inflammatory response, generating a host of reactive lipid products including leukotrienes, prostaglandins, prostacyclins and thromboxanes. Curcumin has been shown to decrease the metabolism of arachadonic acid by downregulating the activity of LOX and COX-2, both at the transcriptional level as well as via post-translational enzyme inhibition [70-72]. More recently, it has been demonstrated that curcumin inhibits prostaglandin E2 biosynthesis through direct inhibition of the microsomal prostaglandin E2 synthase-1 enzyme [73]. In addition, the free-radical scavenging activity of curcumin also contributes to its anti-inflammatory properties by decreasing the amount of oxidative stress that can trigger the inflammatory cascade. The anti-inflammatory properties of curcumin have been investigated in a number of disease entities such as Alzheimer's disease, cardiovascular disease, diabetes, asthma, inflammatory bowel disease, arthritis, pancreatitis and renal disease [74-90].

### Anti-cancer Activity: Suppression of Carcinogenesis

**Figure 2:**
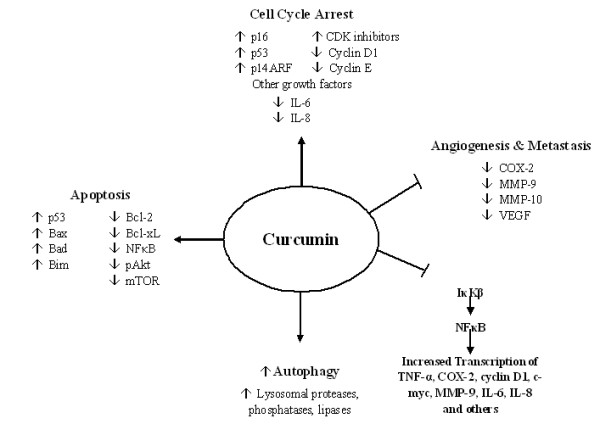
**Overview of the anti-cancer effects of curcumin**. Curcumin suppresses the activation of NF-κB via inhibition of IκKB activity, leading to suppression of many NF-κB-regulated genes involved in tumorigenesis including TNF,COX-2, cyclin D1, c-myc, MMP-9 and interleukins. Curcumin is involved in cell cycle control and stimulation of apoptosis via upregulation of p16 and p53. In addition, curcumin is a modulator of autophagy and has inhibitory effects on tumor angiogenesis and metastasis via suppression of a variety of growth factors including VEGF, COX-2, MMPs and ICAMs.

Curcumin has been studied in multiple human carcinomas including melanoma, head and neck, breast, colon, pancreatic, prostate and ovarian cancers [45,48,53,91-96]. Epidemiological studies attribute the low incidence of colon cancer in India to the chemopreventive and antioxidant properties of diets rich in curcumin [97]. The mechanisms by which curcumin exerts its anti-cancer effects are comprehensive and diverse, targeting many levels of regulation in the processes of cellular growth and apoptosis. Besides the vertical effects of curcumin on various transcription factors, oncogenes and signaling proteins, it also acts at various temporal stages of carcinogenesis----from the initial insults leading to DNA mutations through the process of tumorigenesis, growth and metastasis (Figure 2). Because of the far-reaching effects and multiple targets of curcumin on the cell growth regulatory processes, it holds much promise as a potential chemotherapeutic agent for many human cancers.

Curcumin's potent anti-oxidant and free-radical quenching properties play an important role in the inhibitory effects of the compound on the initial stages of carcinogenesis. It has been shown that curcumin has the ability to suppress UV irradiation-induced DNA mutagenesis and induction of cellular SOS functions [98]. In addition to the inhibitory effects on the production of nitric oxide (NO) and the ability to scavenge DNA-damaging superoxide radicals, curcumin also affects both the Phase I and Phase II enzymes of the hepatic cytochrome p450 enzyme system involved in the oxidation and detoxification of toxic substances. Curcumin has been shown to inhibit the Phase I enzymes (including cytochrome p450 isoforms and p450 reductase) which are induced in response to toxin exposure and create a host of carcinogenic metabolites that contribute to DNA adduct formation during the oxidation of such substances [99]. Conversely, curcumin induces the Phase II enzymes involved in detoxification of toxic metabolites (including glutathione S-transferase, glutathione peroxidase and glutathione reductase) [100]. Curcumin's inhibitory effect on carcinogenesis has been demonstrated in several animal models of various tumor types including oral cancer, mammary carcinoma and intestinal tumors [101-103].

### Effect on Transcription Factors NF-κB and AP-1

NF-κB and AP-1 are two transcription factors intimately involved in the cellular pathways leading to tumorigenesis. As discussed earlier, NF-κB expression is induced by various stressful stimuli (including oxidative stress, cytokines such as TNF-α and IL-1, UV irradiation and infectious antigens) and results in expression of genes involved in inflammation and cellular proliferation. AP-1 is a transcription factor composed of dimers or heterodimers from the Jun, Fos, and ATF families of proteins that binds at a specific DNA site known as TPA-responsive elements (TREs) containing the consensus sequence 5'-TGAG/CTCA-3' [104]. AP-1 is also activated in response to tumor promoters, pro-inflammatory cytokines, UV irradiation and oxidative stress, and the activation of both transcription factors was shown to be required for maintaining transformed tumorigenic phenotypes in mouse epidermal JB6 cells [105]. Like NF-κB, AP-1 regulates the expression of a variety of genes involved in control of the cell cycle and apoptosis. The c-Jun domain of AP-1 is a positive regulator of cyclin D1 expression, contributing to the induction of a tumorigenic phenotype. c-Jun is also a negative regulator of p53 expression and fibroblast cells expressing constitutive levels of c-Jun express lower levels of both p53 and the p53-regulated cyclin-dependant kinase inhibitor p21^Cip1/Waf1 ^and fail to undergo growth arrest following UV exposure [106]. In addition, c-Jun has been shown to down-regulate the expression of p16 and antagonize JunB mediated p16 activation [107].

Curcumin has an inhibitory effect on both NF-κB and AP-1 activation. As reviewed above, curcumin's effect on NF-κB is mediated through inhibition of IκK and results in inactive NF-κB remaining bound to IκBα in the cytoplasm. As a result, curcumin has been shown to suppress the expression of a variety of NF-κB regulated gene products involved in carcinogenesis and tumor growth including cyclin D1, VEGF, COX-2, c-myc, Bcl-2, ICAM-1 and MMP-9 [108,109]. In the case of AP-1, curcumin has been shown to downregulate the production of c-jun mRNA in vivo. Thus, it is proposed that decreased production of the Jun-Fos complex is the underlying mechanism for the decreased AP-1 transcriptional activity in the presence of curcumin and unlike with other inhibitors of Ap-1, this effect is independent of the redox status of the cell [110].

### Effect on Cell Cycle Regulation and Apoptosis

**Figure 3:**
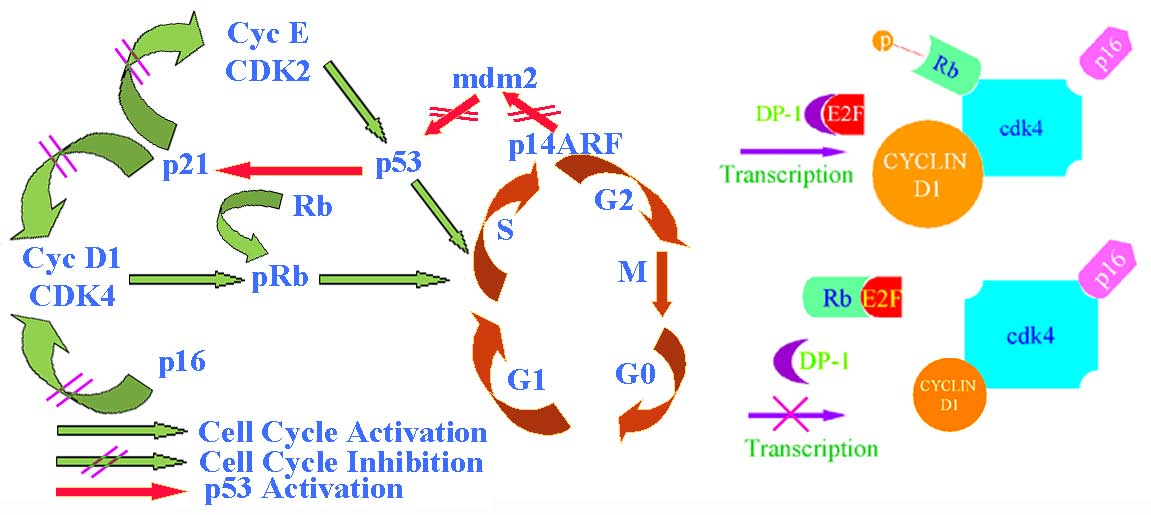
**Cell cycle regulation by Rb and p53 tumor suppressor proteins**. A) Inactivation of Rb and p53 proteins occurs by phosphorylation for the progression of the cell cycle from the G1 to the S phase. Kinase function of CDK4 is activated by cyclin D1 and inactivated by p16 proteins. Cyclin E and p21 control the activation and inactivation of CDK2, respectively. Ubiquitination of p53 takes place by complexing with MDM2 that is blocked by p14ARF (ARF, alternative reading frame). B) Inactivation of p16 by deletion, methylation, or mutation and/or by amplified expression of cyclin D1 leads to increased phosphorylation of Rb resulting in the activation of E2F mediated transcription. However, increased expression of p16 and reduced expression of cyclin D1 results in hypophosphorylated Rb binding to E2F transcription factor leading to the inactivation of transcription.

Cellular growth and proliferation is a highly regulated event in normal cells, and derangements of the cell cycle can lead to uncontrolled proliferation and contribute to the malignant phenotype of tumor cells. The mammalian cell cycle consists of four main stages: G1, S, G2 and M, with G1 and G2 being referred to as "gap" phases between the events of DNA synthesis and mitosis, respectively (Figure 3) [111]. In addition, there is a fifth phase, referred to as G0 which represents a state of quiescence outside the cell cycle in which cells are not actively dividing or preparing to divide. Control of the cell cycle is accomplished via the coordinated interaction of cyclins with their respective cyclin-dependant kinases (CDKs) to form active complexes and drive cells into the next phase at the appropriate time. Regulatory checkpoint control is mediated by the tumor suppressor gene p53 and serves to monitor the accuracy of vital events such as DNA replication and chromosome segregation, and can result in halting of the cell cycle to allow for repair if delinquencies are detected [112].

Specific cyclin/CDK complexes regulate various points in the cycle and are themselves substrates for other regulatory molecules with the most regulated points being the G1/S and G2/M transitions. Cyclin D family of proteins complex with CDK4 and CDK6 and promote progression through G1, Cyclin E/CDK2 complex promotes entry into S phase, Cyclin A/CDK2 stimulates G2 progression, and Cyclin B/CDK1 activates entry into the mitotic phase [113].

There are two distinct families of CDK inhibitors: the INK-4 family (p15^Ink4b^, p16 ^Ink4a^, p18 ^Ink4c^, p19 ^Ink4d^) and the Cip/Kip family (p21 ^Cip1/Waf1^, p27 ^Kip1^, p57 ^Kip2^). The INK4 family of inhibitors binds to CDK4 and CDK 6 to prevent their association with Cyclin D, while the Cip/Kip family binds both CDKs and cyclins to inhibit the formation of the various cyclin/CDK complexes [114,115]. At the G1/S transition, the Cyclin D/CDK4,6 complexes promote progression by phosphorylating the pRB protein, releasing the transcription factor E2F and freeing it to transcribe genes required for cell division [116,117]. Curcumin has been shown to upregulate the expression of the Cip/Kip family of CDK inhibitors (p21 ^Cip1/Waf1 ^and p27 ^Kip1^), thus inhibiting the association of cyclin D1 with CDK4,6 [118]. In addition, decreased phosphorylation of Rb and suppressed transcription of E2F-regulated genes in the presence of curcumin has been demonstrated [119].

Derangements in the regulatory control of the cell cycle can result in the formation of tumor cells, in which growth and proliferation proceeds unchecked. One such example is overexpression of cyclin D1, which has been observed in many types of cancer including hematologic malignancies and various solid tumors [120,121]. Curcumin has been shown to suppress the expression of cyclin D1 in many types of cancer including head and neck, colon, bladder, breast, cervical and pancreatic carcinomas, an effect attributed to curcumin's inhibition of NF-κB activation and subsequent suppression of downstream gene products [48,53,108,109,122-124].

**Figure 4:**
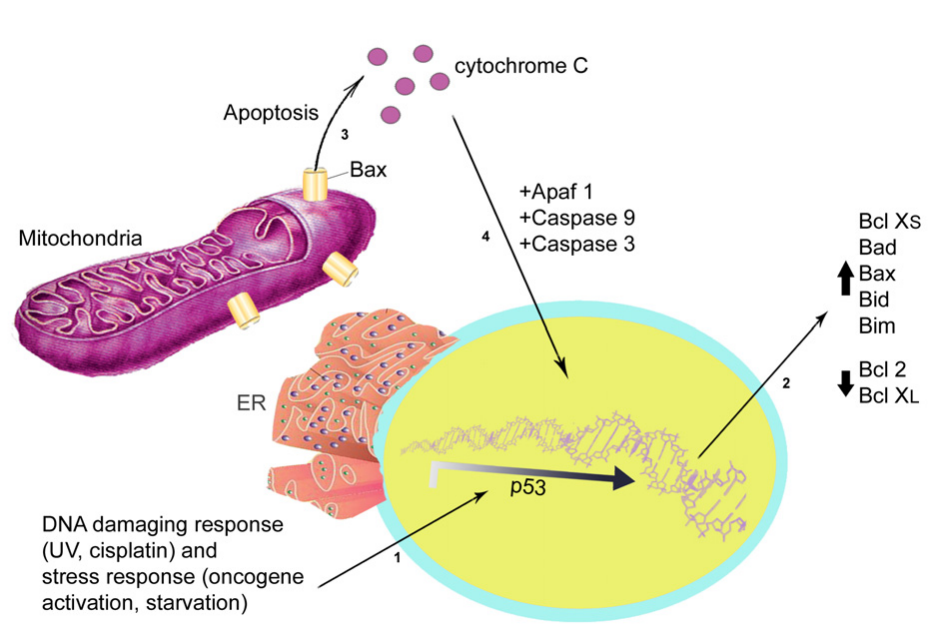
**Programmed cell death type I: Apoptosis: In response to cellular damage, activation of p53 results in increased expression of Bax and antagonism of the anti-apoptotic protein Bcl-2**. As a result, mitochondrial membrane permeability increases and cytochrome C is released into the cytosol. Cytochrome C binds Apaf-1 to form an apoptosome complex leading to activation of caspase-9 and initiation of the caspase cascade and resultant cell death.

There are two main apoptotic pathways: the intrinsic (mitochondrial) pathway and the extrinsic (death receptor) pathway. The intrinsic pathway involves p53 functioning as a transcription factor to upregulate the expression of Bax (Figure 4). Bax is a pro-apoptotic protein that antagonizes Bcl-2, an anti-apoptotic protein that is present in the mitochondrial membrane [125]. When the Bax/Bcl-2 ratio is increased, the protective effect of Bcl-2 on the mitochondrial membrane is disrupted and permeability increases, allowing cytochrome c to leak into the cytosol. Cytochrome c binds to Apaf-1 (apoptotic protease activating factor-1) to form an apoptosome complex, which inititates the caspase cascade via activation of caspase-9 and results in cell death via enzymatic destruction of cytoplasmic proteins and DNA [126]. Curcumin has been shown to selectively induce apoptosis in tumor cells at the G2 phase via upregulation of p53 expression and initiation of the mitochondrial apoptotic pathway via increased Bax expression and cytochrome c release [125-129].

Curcumin also has a stimulatory effect on the extrinsic apoptotic pathway, which is triggered by the binding of "death activators" such as TNF-α, and Fas Ligand to their corresponding cell surface receptors. Activation of these receptors results in activation of caspase-8 via the receptor-attached FADD adapter molecule and initiation of the caspase cascade [130,131]. Curcumin has been shown to increase the levels of Fas and FADD and induce apoptosis in mouse-rat retinal ganglion cells [131]. A study in melanoma demonstrated that curcumin led to apoptosis by promoting aggregation of Fas receptors and by increasing levels of caspase-8 and -3 without altering the level of caspase-9 (unique to the intrinsic pathway) [132]. In addition, curcumin's suppression of the NF-κB mediated cell survival pathway is also important in the compound's pro-apoptotic effect [132-134].

### Effects on Autophagic Cell Death

**Figure 5:**
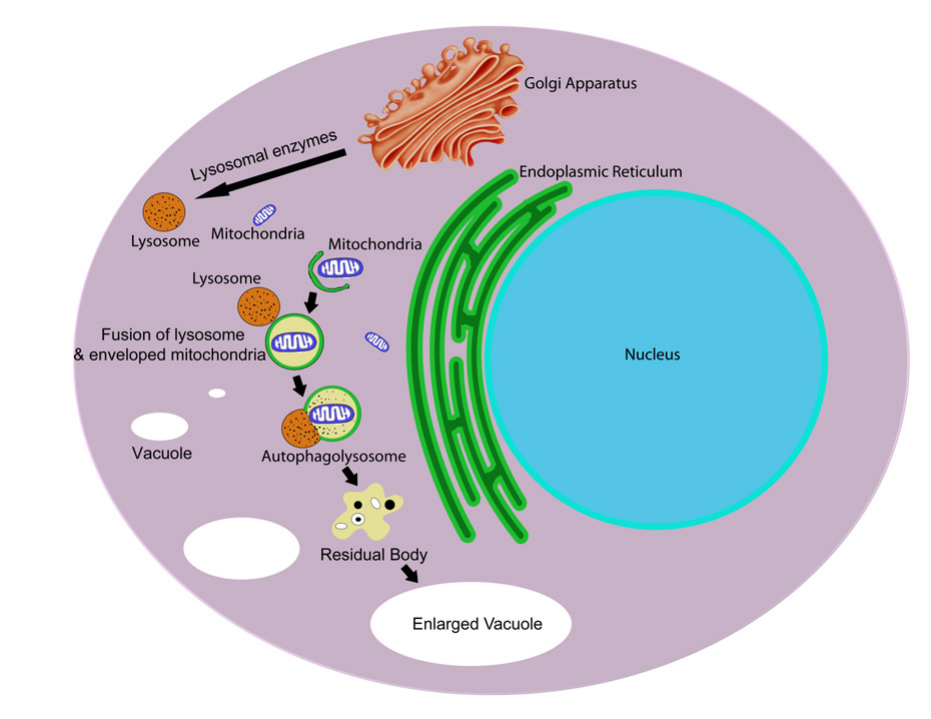
**Programmed cell death type II: Autophagy is a catabolic process by which cells degrade their own components via the lysosomal system**. In response to cellular or nutrient stress, double-layered autophagosomes containing cytoplasmic proteins and organelles are formed following envelopment by a membrane derived from the endoplasmic reticulum. Upon fusion with lysosomes, the contents of these autophagolysosomes are degraded. Autophagy is important as a housekeeping function to promote cell survival and may also function as a pathway of programmed cell death.

Autophagy is a catabolic process in which cells break down their own components via engulfment in vacuoles and degradation through the lysosomal system (Figure 5). The hallmark of autophagy is thus the formation of these so-called "autophagosomes", double layered vacuoles which contain cytoplasmic proteins and organelles targeted for degradation upon fusion with the lysosome [135]. Autophagy is a housekeeping process by which cells may dispose of old or damaged cytoplasmic organelles and proteins, and also serves an adaptive function under conditions of nutrient stress by allowing cells to recycle endogenous biosynthetic substrates such as amino acids.^133 ^In addition to promoting cell survival and function, autophagy is also a method by which cells may undergo programmed cell death [136]. Autophagy is considered Type II programmed cell death (apoptosis is type I and necrosis is type III) and, thus has come under interest as a potential process that may be exploited in the development of anti-cancer chemotherapeutics.

The possible roles of autophagy in carcinogenesis as well as tumor regression in response to therapy are still being elucidated, with seemingly conflicting studies suggesting that induction of autophagy enhances cell death in certain tumor types while mediating chemotherapeutic resistance in others. On one hand, there is evidence that autophagy may be employed by cancer cells to facilitate growth under the stressful metabolic conditions commonly encountered in the tumor microenvironment (such as hypoxia and decreased availability of glucose and other nutrients due to poor vascularization) [137-139]. In addition, the induction of autophagy as an adaptive response mediating resistance to chemotherapy has been observed in multiple tumor types including malignant gliomas, lymphoma, breast, lung and hepatocelluar carcinomas [140-146].

On the other hand, there is genomic evidence that disruption of autophagy is associated with tumorigenesis, as suggested by the mono-allelic deletion of the autophagy-related gene beclin-1 in a high percentage of breast and ovarian cancers [147]. Studies in mice have demonstrated that monoallelic deletion of beclin-1 increases the frequency of spontaneous malignancies including hepatocellular carcinoma, B cell lymphoma and lung adenocarcinomas in beclin-1 +/- mice, suggesting beclin-1 as a haploinsufficent tumor suppressor gene [148]. In addition it was found that monoallelic deletion of beclin-1 resulted in increased cellular proliferation, decreased autophagy as measured by expression of the autophagosome membrane protein LC3, and accelerated the development of hepatitis B-induced premalignant lesions [149]. Conversely, transfection of beclin-1 into MCF-7 breast cancer cells (which express a very low baseline level of the protein) inhibited the cellular proliferation and tumorigenicity in a nude mouse xenograft model [147].

While autophagy appears to play a role in mediating chemoresistance in certain cancers as described above, there is also data supporting that autophagy may also induce non-apoptotic cell death in response to chemotherapy. In human (MCF-7) estrogen-receptor positive breast cancer, both tamoxifen and paclitaxel were found to induce autophagic cell death in cell culture [150,151]. Arsenic trioxide was found to induce autophagic cell death in malignant glioma, leukemia and fibrosarcoma cells, and in leukemia this effect was accompanied by up-regulation of beclin-1 [152-154]. The small molecule tyrosine kinase inhibitor imatinib has been shown to induce cellular autophagy, an effect that may sensitize drug-resistant Kaposi sarcoma cells [155,156]. Interestingly, imatinib's induction of autophagy seems to decrease its effectiveness in chronic myelogenous leukemia (CML) and blocking of autophagy lead to increased apoptotic cell death [142]. Studies done in malignant glioma cells have also yielded varying results; while autophagy induced by arsenic trioxide resulted in increased cell death, treatment with temolozamide and etoposide led to an increase in ATP that exerted a protective effect [152,157]. Likewise, there is controversy regarding the effect of autophagy induction on radiation sensitivity. Studies in breast cancer have suggested that vitamin D-dependant radiosensitization is mediated through autophagy, while autophagy has demonstrated both radiosensitizing and dampening effects in malignant gliomas [158-160].

Curcumin has been shown to be an inducer of autophagic cell death in chronic myelogenous leukemia, esophageal cancer and malignant glioma cells [161-163]. In malignant glioma cells, curcumin induced G2/M cell cycle arrest and non-apoptotic autophagic death. This effect was mediated through curcumin's inhibition of the Akt/mTOR/p70S6 kinase pathway and inhibition of the ERK1/2 pathway, which are both involved in the regulation of autophagy induced by nutrient stress. In addition, these effects were confirmed via activation of the Akt/mTOR/p70S6 pathway which decreased curcumin-induced autophagic cell death as well as activation of the ERK1/2 pathway, which resulted in inhibition of autophagy and induction of apoptosis [163,164]. While the current data on autophagy and cancer is far from providing a consensus, it is evident that regulation of this process may play an important role in tumorigenesis and response to therapy thus making pharmacologic modulators of autophagy attractive candidates for further study.

### Effects on Angiogenesis and Metastasis

The stimulation of new blood vessel growth is an essential step for tumor growth and metastasis in order to provide for the metabolic needs of rapidly proliferating malignant cells. Angiogenesis is regulated by a variety of pro-angiogenic genes and signaling molecules including vascular endothelial growth factor (VEGF), basic fibroblast growth factor (bFGF), epidermal growth factor (EGF), platelet-derived growth factors, hypoxia-inducible factors, angiopoetin-1 and 2, and matrix metalloproteinases [165]. The role of angiogenesis in tumor growth has been targeted by newer chemotherapeutic agents such as bevacizumab, an anti-VEGF monoclonal antibody that is FDA approved for metastatic forms of various cancers including colon, non-small cell lung, HER2-negative breast cancer and renal cell carcinoma. In addition to bevacizumab, sorafenib and sunitinib are novel small-molecule inhibitors of multiple receptor tyrosine kinase (RTK) pathways involved in signal transduction from angiogenic receptors such as VEGFR and PDGF-R that are approved for renal cell carcinoma as well as hepatocellular carcinoma and gastrointestinal stromal tumor, respectively [166]. Curcumin has demonstrated an anti-angiogenic effect in vivo in xenograft models of various tumors including glioblastoma, hepatocelluar carcinoma, prostate and ovarian carcinomas [134,167-169]. Curcumin has been shown to regulate a variety of pro-angiogenic growth factors, enzymes and transcription factors including bFGF, VEGF, angiopoetin-1 and 2, COX-2, matrix metalloproteinase-9 (MMP-9), AP-1 and NF-κB [170-172]. Curcumin has also been shown to inhibit the angiogenic response to FGF-2 stimulation in mouse endothelial cells and decrease the expression of MMP-9, an enzyme involved in tissue remodeling that is important for the growth of new blood vessels [171]. In addition, curcumin treatment decreased the levels of the angiogenic biomarkers COX-2 and VEGF in hepatocelluar carcinoma cells, and resulted in a reduction in tumor neocapillary density compared to the untreated cells [172].

In addition to its inhibitory effects on angiogenesis, curcumin has also been demonstrated to affect a number of cellular adhesion molecules involved in the processes of tumor growth and metastasis. A study of curcumin in metastatic melanoma demonstrated a dose dependant reduction in binding to extracellular matrix proteins, decreased expression of alpha5beta1 and alpha(v)beta3 integrin receptors and increased expression of various anti-metastatic proteins including tissue inhibitor metalloproteinase (TIMP-2), nonmetastatic gene 23 (Nm23) and E-cadherin [173]. E-cadherin expression is important in maintaining the integrity of intercellular adhesion though binding to various catenins (including β-catenin), and loss of E-cadherin is associated with an increased tendency for tumor metastasis [174]. Anti-metastatic effects of curcumin have also been demonstrated in the MDA-MB-231 breast cancer cell line, resulting in decreased expression of matrix metalloproteinases, ICAM-1 and chemokine receptor 4 (CXCR4) and suppressed cell migration and invasion [175]. In addition, curcumin was shown to decrease the ability of paclitaxel-resistant breast cancer cells to form lung metastases via suppression of various anti-apoptotic proteins (including XIAP, Bcl-2 and IAP1 and 2), proliferative (COX-2, c-myc and cyclin D1), and metastatic proteins (MMP-9, VEGF and ICAM-1) [176]. As many pro-angiogenic and pro-metastatic genes are regulated by NF-κB (including COX-2, VEGF, ICAM-1 and MMP-9 among others), curcumin's suppressive effect on NF-κB activation likely plays a key role in mediating the compound's anti-angiogenic and anti-tumorigenic effects.

### Therapeutic Activity of Curcumin in Head and Neck Squamous Cell Carcinoma

Curcumin has been studied in various in vitro and vivo models of head and neck squamous cell carcinoma with promising results. An overview of current literature supporting the spice's utility in the treatment of head and neck cancer including as a chemopreventive agent, as well as future directions for study is presented below.

### In Vitro Studies

Studies of curcumin in various head and neck cancer cell lines have demonstrated decreased cell growth and survival, concomitant with the compound's effects on molecular pathways involved in cellular proliferation. Expression of constitutively active NF-κB and IκK has been observed in multiple oral squamous cell carcinoma cell lines, and curcumin treatment was shown to suppress growth and survival of these cell lines via inhibition of NF-κB activation [43]. Signal-transducer-and-activator-of-transcription-3 (STAT3) is a signaling protein observed to be overexpressed in multiple head and neck cancers, and curcumin was shown to suppress the IL-6 mediated phosphorylation of STAT3 as well as inhibiting nuclear localization [177]. In another study, Chakravarti et al [178] demonstrated that curcumin suppressed the growth of immortalized oral mucosal epithelial cells and squamous cell carcinoma cells (UMSCC22B and SCC4) while having minimal effect on normal oral epithelial cells. Curcumin was shown to reduce the efficiency of the eIF4F translational complex of these immortalized cells via suppression of phosphorylation of 4E-BP1, eIF4G, eIF4B and Mnk1, as well as a reduction in the total levels of eIF4E and Mnk1. In SAS oral cancer cells, curcumin induced the promoter activity of insulin-like growth factor binding protein-5 (IGFBP-5) and CCAAT/enhancer-binding protein alpha (C/EBPalpha), proteins involved in the suppression of head and neck cancers. The inhibitory effects of curcumin on IGFBP-5 and C/EBP-alpha were mediated via p38 activation, and resulted in decreased in vivo tumorigenesis in a mouse xenograft model [179].

Our laboratory has studied the effects of curcumin in several head and neck squamous cell carcinoma cell lines: CCL23 (laryngeal), CAL27, UM-SCC14A and UM-SCC1 (oral) [48,53,180]. The growth suppressive effect was shown to be mainly mediated via the effects of curcumin on the NF-κB pathway. Curcumin was shown to decrease the expression of NF-κB and also inhibited its nuclear localization; this observation was supported by a concomitant decrease in phospho-IκB-α expression [46]. In addition, the expression levels of multiple NF-κB regulated gene products (including cyclin D1, MMP-9, COX-2, Bcl-2, Bcl-xL, Il-6, IL-8, Mcl-1L and Mcl-1S) were reduced [48,53].

It has been demonstrated that the curcumin-induced suppression of the NF-κB pathway in head and neck cancer cells is due to inhibition of IκK (inhibitor kappa B kinase), thus blocking the phosphorylation of IκB-α and resulting in NF-κB sequestration in the cytoplasm. We have shown dose-dependent suppression of IL-6 and IL-8 following curcumin treatment in CCL23, CAL27, UM-SCC1 and UM-SCC14A cell lines via inhibition of IκK activity [178]. Furthermore, the curcumin-induced inhibition of IκK was found to take place via an AKT-independent mechanism [53]. The data on curcumin's effect on the AKT pathway is varying; while it has been shown to act independently of AKT in HNSCC as well as melanoma, curcumin suppresses the AKT pathway in other tumors such as malignant gliomas and pancreatic cancer [96,163,181]. AKT (another kinase of transcription, also known as protein kinase B) is a protein kinase involved in signal transduction from oncogenes and growth factors. The AKT signaling cascade is stimulated by EGFR, and represents one pathway by which NF-κB may be activated [182]. As discussed earlier, EGFR is overexpressed in many head and neck cancers and molecular therapies targeting the EGFR/AKT signaling cascade (such as cetuximab) have been shown to increase the therapeutic efficacy of standard platinum-based chemotherapy [16,17]. The finding that curcumin suppresses NF-κB independently of the AKT pathway in HNSCC is of clinical significance, as it acts via a different mechanism than cetuximab and the two agents could potentially be used in combination for treating head and neck cancers.

### In vivo studies

Curcumin has demonstrated *in vivo *growth suppressive effects on head and neck squamous cell carcinoma using nude mouse xenograft models. The lipophilic nature of curcumin and relative insolubility in aqueous solutions, combined with short half life and low bioavailability following oral administration has presented a significant challenge in developing an effective delivery system for its use as a chemotherapeutic agent [183]. In an effort to overcome this obstacle, various strategies are being tried including the use of piperine as an adjuvant agent to slow curcumin breakdown as well as the development of liposomal, phospholipid and nanoparticulated formulations of the compound to enable intravenous administration [183]. Liposomal formulations of curcumin have been studied in various cancers including pancreatic, colorectal and prostate [184-186].

Intravenous liposomal curcumin has been studied by our laboratory in mouse xenograft tumors of the oral cancer cell lines CAL27 and UM-SCC-1, and was found to be both nontoxic as well as effective at suppressing tumor growth. Xenograft mouse tumors were stratified into groups receiving no treatment, treatment with empty liposomes or treatment with liposome encapsulated curcumin and a statistically significant growth suppressive effect was observed in the liposomal curcumin group [53]. The presence of curcumin in mouse serum and liver was confirmed using liquid chromatography-mass spectrophotometry, demonstrating increased systemic absorption of liposomal curcumin relative to a DMSO-suspension of curcumin. Immunohistochemistry of the tumor samples revealed decreased expression of NF-κB in the liposomal curcumin-treated tumors relative to both the liposomal control and untreated groups, while the staining intensity of pAKT did not show a significant difference among the three treatment groups, further supporting the in vitro findings that curcumin's growth suppressive effects are related to the suppression of NF-κB in an AKT-independent manner [53].

A recent study by Clark et al [187] has shown chemopreventive effects of curcumin in mouse xenograft models of oral squamous cell carcinoma. These authors have demonstrated an inhibitory effect on tumor growth following treatment of the mice with an oral curcumin solution both prior to inoculation of SCC40 tongue squamous cell carcinoma cells as well as when curcumin was initiated after tumor formation. In addition, curcumin oral solution was studied in a mouse model of oral carcinogenesis in which the tobacco derivative 4-nitroquinolone-1-oxide was painted inside the animal's mouths several times weekly, with or without concurrent oral curcumin solution. In the mice receiving concurrent curcumin, tumor-free and overall survival times were significantly increased. Here, the effects of cucumin on carcinogenesis suppression were found to be mediated via inhibition of the AKT/mTOR pathway following analysis of treated and untreated tumor cell extracts. Finally, curcumin was found to decrease migration and invasion of malignant oral squamous cells via a downregulation in MMP-9 expression.

### Curcumin as an adjuvant therapy

While studies of curcumin as a single agent in the treatment of head and neck cancer have shown promising results, there is significant interest in potentially using the compound as an adjuvant agent in combination with standard platinum-based chemotherapy for the treatment of head and neck tumors. Data from our laboratory in both CAL27 and UMSCC-1 cell lines demonstrated an increased growth suppressive effect in cells treated with a combination of liposomal curcumin and cisplatin, both in vitro as well as in mouse xenograft tumor models [188]. While treatment with either curcumin or cisplatin in vitro resulted in cell death, a combination of curcumin and suboptimal concentrations of cisplatin demonstrated a significant growth suppressive effect compared to treatment with either agent alone. Curcumin's suppressive effect was again shown to derive from the inhibition of cytoplasmic and nuclear IκK, leading to inhibition of NF-κB activity. There was no effect on pAKT, supporting an AKT-independent mechanism for NF-κB inhibition. Cisplatin treatment led to cellular senescence, an effect mediated through increased expression of p16 and p53 [7]. Differing mechanisms of curcumin and cisplatin suggest potential for the clinical use of subtherapeutic doses of cisplatin in combination with curcumin to accomplish effective suppression of tumor growth while minimizing cisplatin's toxic side effects.

In addition to the potential synergistic effect of curcumin with platinum based chemotherapy, the spice may also have potential utility as an enhancer of radiation therapy. A recent study by Khafif et al [189] compared the effects of curcumin and single-dose radiation alone and in combination in the HNSCC cell lines SCC-1, SCC-9, A431 and KB. In vitro growth suppression with either curcumin or radiation was observed in all four cell lines, and the combination of both therapies resulted in an additive growth suppressive effect. Curcumin was found to arrest carcinoma cells in the G2/M phase of the cell cycle, in which cells are more susceptible to the cytotoxic effects of radiotherapy. In addition, curcumin was shown to decrease COX-2 expression and inhibit EGFR phosphorylation in SCC-1 cells. In vivo experiments using orthotopic mouse models of SCC-1 tumors also supported the additive effects of curcumin and radiation therapy. While curcumin has exhibited varying effects on radiation sensitivity in different cancer cell types, its effect as a radiosensitizer has been supported in several other tumors in addition to HNSCC including prostate, colorectal and ovarian cancers [190-193].

### Future Directions of Study for Curcumin in HNSCC

As discussed in this review, curcumin has demonstrated powerful anti-cancer effects in a variety of malignancies via its effects on a host of biological pathways involved in tumorigenesis and cellular growth. Continuing investigation into the molecular pathways affected by curcumin is indicated to further define the effects of the compound on growth signaling pathways, apoptotic and non-apoptotic cell death, oncogene and tumor suppressor regulation. Translating this abundance of molecular data into eventual clinical applications is a paramount goal of future research. In head and neck cancers, a promising area of study centers around the use of curcumin to treat platinum-refractory tumors, which are associated with a poorer prognosis and have a tendency for disease recurrence. Some work has been done in the area of characterizing putative 'cancer stem cells' in a variety of tumor types including HNSCC, breast, colorectal and prostate cancers [194-197]. Cancer stem cells are not true multipotent stem cells, but are a sub-population of highly tumorigenic cells that are theorized to contribute to chemoresistance and recurrence [198]. CD44 is a cell surface marker that has been shown to be highly expressed in putative head and neck cancer stem cells [196-200]. Our laboratory has demonstrated a population of CD44^High ^putative stem cells within the UM-SCC1 cell line that possess increased tumorigenicity, growth rate and resistance to cisplatin treatment relative to CD44^Low ^cells [201]. Investigating the growth suppressive properties of curcumin in these highly tumorigenic cells could be an initial study in quantifying the utility of the compound in chemotherapy-resistant head and neck cancers, and may have applications in other types of chemoresistant malignancies as well. The solubility of curcumin in an intravenous delivery system is a major consideration in formulating the compound as a suitable chemotherapeutic agent. While our laboratory and others have shown increased therapeutic efficacy of liposomal curcumin, it is possible that other delivery systems may yield superior bioavailability and therapeutic results.

### Other Biologic Therapies Currently Under Investigation in HNSCC

In addition to curcumin, multiple other biologically targeted agents are currently being studied in head and neck cancer. As previously mentioned EGFR is overexpressed in many head and neck cancers and therefore represents a promising potential therapeutic target. The anti-EGFR monoclonal antibody cetuximab is approved both in combination with radiotherapy as well as a single agent for platinum-resistant HNSCC, but is also being investigated in combination with standard chemotherapeutic regimens. A Phase I/II trial of cetuximab with 5-FU and either carboplatin or cisplatin showed increased survival without a significantly increased toxicity relative to the standard chemotherapy regimen (Table 1) [202]. However, acquired resistance to EGFR inhibition by cetuximab has emerged as a therapeutic challenge [203]. A variant of EFGR (EGFRvIII) that results in constitutive activation of the downstream Ras/Raf/MAPK, STAT3 and PI3/AKT/mTOR pathways has been observed in HNSCC that is not responsive to EGFR inhibition [204]. In addition to targeting the extracellular EGF receptor, tyrosine kinase inhibitors (erlotinib, gefitinib, lapatinib) that block intracellular EGFR phosphorylation and inhibit downstream signal transduction are also being studied in HNSCC. A Phase II study of erlotinib in patients with recurrent or metastatic head and neck cancer showed an increase in disease stabilization (Table 2) [205]. A Phase III trial of gefitinib alone compared to methotrexate monotherapy in recurrent HNSCC failed to show a significant survival increase, but a more recent study of gefitinib added to concurrent chemoradiation showed a favorable response that correlated to the number of EGFR copies in the various tumors [206,207].

**Table 2:** Current molecular pathway based therapies in head and neck cancer

Agent	Molecular target	Phase/model of investigation in HNSCC	Reference
Erlotinib/Gefitinib/Lapatinib	Anti-EGFR receptor kinase	Erlotinib: Phase II study in refractory/metastatic HNSCC Gefatinib: PhaseIII vs methotraxate in recurrent HNSCC	[205-207]
Bevacizumab	Anti-VEGF monoclonal antibody	Mouse xenograft models: in combination with paclitaxel Phase I/II trial of bevacizumab and erlotinib in recurrent/metastatic HNSCC	[208]
Rapamycin derivatives (Everolimus, Deferolimus Temserolimus)	Inhibition of PI3K/AKT/mTOR pathway	Mouse xenograft model: Single agent CCI-779 in minimal residual disease Mouse xenograft model: CCI-779 in combination with radiotherapy	[210-212]
Sorafenib	Multikinase inhibitor targeting MAP kinase, VEGFR, PDGFR FLT3, Ret, c-kit	Phase II: in chemotherapy-naïve persistent/recurrent HNSCC	[213,214]
Pemetrexed	Folate antimetabolite	Phase I: in combination with cisplatin in HNSCC Phase II: Pemetrexed plus gemcitabine in recurrent/metastatic HNSCC	[215,216]
Bortezomib	Proteosome inhibitor	Phase I: Bortezomib with re-irradiation in HNSCC Phase II: Bortezomib with docetaxel in recurrent/metastatic HNSCC in vitro: Synergistic anti-tumor effect of Bortezomib and cisplatin in HNSCC cell lines	[217-219]
Curcumin	Inhibition of NF-kB activation, Suppression of interleukins, Cell cycle inhibition, Suppression of VEGF and other angionegic factors, Up-regulation of cellular adhesion molecules, inhibition of STAT 3	in vitro: Growth suppression of HNSCC cell lines with decreased NF-kB activation	[48,53]
		in vitro: Suppression of IL-6 and IL-8 expression in HNSCC	[180]
		in vitro: Growth suppression of immortalized epithelial cells	[178,187]
		Mouse xenograft: Suppression of CAL 27 cell line tumors	[48,53]
		Mouse model: Inhibition of oral carcinogenesis	[178,187]
		in vitro and mouse model: Synergistic anti-tumor effect of Curcumin and cisplatin in CAL 27 and UM-SCC1 cell lines	[188]
		in vitro and mouse model: Radiosensitization of SCC-1, SCC-9, A431, and KB HNSCC cell lines with curcumin	[189,190]

Targeted therapies against the vascular endothelial growth factor receptor (VEGF) are also being evaluated in HNSCC. The anti-VEGF monoclonal antibody bevacizumab in combination with paclitaxel showed increased anti-tumor effects in mouse xenograft tumor models of HNSCC compared to either agent alone [208]. A combination of bevacizumab and erlotinib was also studied in a Phase I/II clinical trial in recurrent or metastatic HNSCC and demonstrated a response rate of 15%, which was significantly increased compared to prior studies of erlotinib (5%) or single agent anti-angiogenic therapies (4%) [209].

Constitutive activation of the PI3-K/Akt/mTOR pathway has been observed in head and neck cancer and is associated with resistance of such tumors to radiation and chemotherapy [210]. As such employing rapamycin derivatives such as everolimus, deforolimus and temserolimus may prove useful in the treatment of refractory head and neck cancers. The experimental mTOR inhibitor CCI-779 has been studied as a single agent in mouse models of HNSCC minimal residual disease and demonstrated a significant increase in the tumor-free rate between the treatment and control groups (50% vs. 4% respectively) as well as a reduction in tumor volume in the CCI-779 group [211]. Analysis of the radiosensitizing effect of CCI-779 in mouse xenograft models of both cisplatin sensitive (FaDu) and resistant (SCC40) head and neck squamous cell carcinoma showed increased survival relative to radiotherapy alone. In addition, the antitumor effects of CCI-779 plus radiotherapy were superior when compared to conventional chemoradiotherapy with cisplatin in both the FaDu and SCC40 xenograft tumors [212].

In addition to the major pathways discussed above, several other novel biologic agents are under investigation in head and neck cancer that merit brief mention. Sorafenib is a multikinase inhibitor targeting the MAP kinase pathway (RAF/MEK/ERK) as well as several other receptor tyrosine kinases including VEGFR, PDGFR, FLT3, Ret and c-kit [213]. It is currently FDA approved for treatment of advanced primary renal cell carcinoma and hepatocellular carcinoma. A Phase II trial of sorafenib in chemotherapy-naïve persistent or recurrent HNSCC showed a relatively poor response rate of 2%, but progression-free and overall survival compared favorably with other single agent Phase II trials [214]. Pemetrexed is a folate antimetabolite currently FDA approved in combination with cisplatin for malignant mesothelioma. A Phase I study of pemetrexed in combination with cisplatin for HNSCC showed no enhancement in cisplatin-related toxicities or alteration of the cisplatin pharmacokinetics [215]. A Phase II trial of pemetrexed plus gemcitabine in recurrent or metastatic HNSCC demonstrated a partial response rate of 16% and was well-tolerated [216]. Bortezomib is a proteosome inhibitor that is FDA approved for the treatment of multiple myeloma and mantle cell lymphoma. The cytoplasmic to nuclear translocation and activation of NF-κB is a proteosome-dependant process, and bortezomib has been shown to inhibit nuclear activation of the RelA and NF-κB1 subunits in HNSCC [217]. In addition bortezomib has been found to induce apoptosis in HNSCC cells via up-regulation of the pro-apoptotic proteins Bik and Bim, and the combination of bortezomib and cisplatin resulted in a synergistic tumoricidal effect in HNSCC [218]. A Phase II trial of bortezomib in combination with docetaxel in recurrent and/or metastatic HNSCC was well-tolerated and demonstrated better therapeutic response in tumors expressing lower levels of NF-κB associated genes [219].

## Conclusions

The need for alternative and less toxic therapies for head and neck squamous cell carcinoma is clear. Multiple molecular pathways such as NF-κB activation, EGFR and PI3/AKT/mTOR signaling, STAT3 expression, the MAP kinase cascade and VEGF-mediated angiogenesis have been shown to be deregulated in HNSCC and represent potential therapeutic targets. While some promising results from such targeted therapies have been obtained, the complexity of interaction between these signaling pathways may contribute to the limited clinical response seen with the use of single-agent biologic therapies. As a natural product, curcumin is both non-toxic as well as diversified in its inhibitory effects on a multitude of pathways involved in carcinogenesis and tumor formation. While the compound alone has shown some anti-tumor effects in HNSCC, curcumin's lack of systemic toxicity and broad-reaching mechanism of action may make it best suited as an adjuvant therapy for head and neck cancers that are resistant to currently available therapies.

## Competing interests

The authors declare that they have no competing interests.

## Authors' contributions

RW carried out literature survey and in association with MSV contributed to the design and draft of the manuscript. MBW participated in the design and coordination of the manuscript. ESS conceived of the study, and participated in the design and drafting of the manuscript. RW and ESS were also involved in the design of the figures. All authors read and approved the final manuscript.

## Credit

Permission is granted for the use of figure 3 from Arch. Otolaryngology Head and Neck Surgery 2006, **132: **317-326 "Copyright (2006) American Medical Association, All rights reserved."
